# Effect of stem cell treatment on functional recovery of spinocerebellar ataxia: systematic review and meta-analysis

**DOI:** 10.1186/s40673-021-00130-8

**Published:** 2021-02-25

**Authors:** Pablo Andrei Appelt, Kristin Comella, Luciane Aparecida Pascucci Sande de Souza, Gustavo José Luvizutto

**Affiliations:** 1 Master student in Physical Therapy of Federal University of Triângulo Mineiro (UFTM), Uberaba, Minas Gerais Brazil; 2 Chief Scientific Officer of US Stem Cell Clinic, Weston, Florida USA; 3 Professor of Applied Physical Therapy Department of Federal University of Triângulo Mineiro (UFTM), Uberaba, Minas Gerais Brazil

**Keywords:** Spinocerebellar ataxia, Stem cells, Hereditary, Mesenchymal

## Abstract

**Background:**

Spinocerebellar ataxia is a hereditary neurodegenerative disease characterized by changes in balance, locomotion and motor coordination. Stem cell therapies are currently being investigated as an alternative to delay the evolution of the disease, and some experimental studies have investigated the effect of stem cell treatment on spinocerebellar ataxia.

**Objectives:**

The aim of this review was to investigate whether the application of stem cells produced an effect on functional recovery in individuals with spinocerebellar ataxia.

**Methods:**

The studies included in this review investigated the efficacy and safety of a protocol for the application of mesenchymal stem cells extracted from umbilical cord and adipose tissue. Two studies used intrathecal route for application and one study used intravenous route.

**Results:**

Studies have shown clinical improvement in the scores of the ICARS (International Cooperative Ataxia Rating Scale), ADL (Activities of Daily Living Scale), BBS (Berg Balance Scale) and SARA (Scale for the Assessment and Rating of Ataxia), but lacked statistical significance.

**Conclusions:**

There was low evidence for recommending stem cell therapy in individuals with spinocerebellar ataxia, and no statistical difference was observed for improving functional recovery of patients. Further studies are needed with different designs, largest sample sizes and placebo control, to fully understand anticipated outcomes of cellular therapy for spinocerebellar ataxia.

## Introduction

The term ataxia is used to describe a neurodegenerative disease with heterogeneous genotypic and phenotypic characteristics [1]. Spinocerebellar ataxias (SCA) are a subset of hereditary cerebellar ataxias that are autosomal dominantly transmitted and has clinical and neuropathologic heterogeneous manifestations [1, 2], caused by degenerative changes in the cerebellum. There are more than 40 types of spinocerebellar ataxias [3], and spinocerebellar ataxia type III (SCA3), also known as Machado Joseph’s disease, is the most prevalent type [4].

There is a wide variety of clinical manifestations in SCA, such as chances in muscle tone, inadequate motor coordination, poor postural control, including changes in locomotion, dysarthria, progressive ophthalmoplegia, extrapyramidal signs including dystonia, stiffness and/or bradykinesia, and changes in the lower motor neuron, with fasciculations, amyotrophy, decreased sensitivity, eyelid retraction, weight loss, sleep disorders and fatigue [5, 6]. Thus, even knowing that there may be partial involvement of the cerebellum, the treatment of degenerative cerebellar diseases is a challenge, because of its progressive nature, generating important changes in balance, coordination, and locomotion, among others. The physical deconditioning and motor impairment, leads to a reduction of skills in activities of daily living, as well as other negative consequences such as falls, immobility, reclusion of social life and worsening quality of life [7, 8].

Many treatments have been considered to reduce the impact of the SCA on functionality, as well as to delay the degeneration of the cerebellum. Among the therapeutic options, multipotent mesenchymal stem cells have been considered in this population due to their immunomodulatory and regenerative properties [9]. These cells can be isolated from bone marrow, adipose tissue, placenta, thymus, umbilical cord and dental pulp, and are increasingly becoming a therapeutic option for several degenerative diseases, as they can generate an exogenous supply of cells capable of promoting neurogenesis and modulatory effects, stimulating plasticity and cell differentiation [10].

Stem cell-based therapies represent a new therapeutic strategy for SCA [2, 11]. In preclinical animal models, positive results have been observed in reducing cerebellar degeneration. Some studies have observed that the stem cell therapy can slow or stop the progression of spinocerebellar ataxias, with improved motor functions [12] and found that intravenous mesenchymal stem cell transplantation delayed the onset of loss of motor function in rats with SCA2 [13]. Another study [14] showed delay in the onset of locomotor deficits and in the degeneration of sensory neurons. Other animal studies observed tissue repair of Purkinje cells and cerebellar interneurons after stem cell transplantation [15].

In addition, neurotransplants have been performed in several models with mutant mice using different cell types and techniques to stop or delay the degeneration of Purkinje cells and restore normal cerebellar architecture [11]. These preclinical studies show promise for the use of stem cells in neurodegenerative diseases, mainly in the SCA, but clinical trials in humans will need to be completed to confirm efficacy [16]. Therefore, due to the lack of studies evaluating the clinical effects of stem cell application in SCA, there is a need for further studies to elucidate the best available evidence on the mechanisms involved in this therapy. The aim of this study was to evaluate the available literature about the effects of stem cell treatments in patients with SCA and its use to reduce motor impairments and improve functionality.

## Material and methods

We adhere to the methods described in *Cochrane Handbook for Intervention Reviews* [17]. Our review also follows the items recommended by the systematic reviews protocol, according to the checklist (PRISMA) [18]. This review was recorded in the International Prospective Registry of Systematic Reviews (PROSPERO CRD42020179245).

### Eligibility criteria

The eligibility criteria were as follows:

- Participants: individuals with spinocerebellar ataxia with clinical and/or neuroimage and/or genetic confirmation. The clinical confirmation was based on Harding’s classification. The clinical setting of symptoms of SCAs include gait ataxia and incoordination, nystagmus/visual problems and dysarthria. In addition, patients can present pyramidal, extrapyramidal signs, ophthalmoplegia and cognitive impairment [1]. Neuroimage confirmation included magnetic resonance imaging (MRI) showing cerebellum and/or brain stem atrophy; and genetic confirmation by molecular tests.
- Interventions: stem cell application protocols; The stem cell application protocols included in this review were with UCMSCs (umbilical cord mesenchymal stem cells) and AD-MSCs (adipose tissue mesenchymal stem cells). All routes of stem cell administration were included.
- Control: any comparison;
- Outcomes: all impairments were considered (motor function, language, ocular motility, dexterity, balance, and locomotion), functional recovery (follow-up) and treatment safety.
- Study design: randomized, quasi-randomized and non-randomized clinical trials.

### Data search

In the virtual search for studies, we used the databases PUBMED, SCIELO, OVID, CINAHL, WEB OF SCIENCE, SCIENCE DIRECT, SPRINGER, PEDRO, LILACS, SCOPUS, COCHRANE and CLINICAL TRIALS through February of 2021. The search strategy was described in the Table 1. All searches were conducted with the assistance of a trained medical librarian. We also searched the reference lists of relevant articles and conference proceedings and contacted the authors of the included trials. There was no language restriction.

**Table 1:** Key Terms and MeSH Strategy Employed During the Literature Review

PICO Process	Keyword/Mesh
(P) Population	*Cerebellar degeneration OR Spinocerebellar Ataxia OR Dominantly-Inherited Spinocerebellar Ataxias OR Dominantly Inherited Spinocerebellar Ataxias OR Dominantly-Inherited Spinocerebellar Ataxia OR Spinocerebellar Atrophies OR Spinocerebellar Atrophy OR Spinocerebellar Ataxia Type 2 OR Type 2 OR Spinocerebellar Ataxia OR Spinocerebellar Ataxia 2 OR Spinocerebellar Ataxia 2 s OR Wadia-Swami Syndrome OR Cerebellar Degeneration with Slow Eye Movements OR Olivopontocerebellar Atrophy II OR Olivopontocerebellar Atrophy IIs OR Spinocerebellar Atrophy II OR Spinocerebellar Atrophy IIs OR Olivopontocerebellar Atrophy 2 OR Olivopontocerebellar Atrophy 2 s OR Spinocerebellar Ataxia with Slow Eye Movements OR Spinocerebellar Atrophy 2 OR Spinocerebellar Atrophy 2 s OR Spinocerebellar Degeneration with Slow Eye Movements OR Wadia Swami Syndrome OR Spinocerebellar Ataxia-2 OR Spinocerebellar Ataxia Type 7 OR Type 7 Spinocerebellar Ataxia OR OPCA with Retinal Degeneration OR Olivopontocerebellar Atrophy III OR Olivopontocerebellar Atrophy IIIs OR Autosomal Dominant Cerebellar Ataxia, Type II OR OPCA with Macular Degeneration and External Ophthalmoplegia OR Spinocerebellar Ataxia-7 OR Spinocerebellar Ataxia 7 OR Spinocerebellar Ataxia 7 s OR Spinocerebellar Ataxia Type 5 OR Type 5 Spinocerebellar Ataxia OR Spinocerebellar Ataxia 5 OR Spinocerebellar Ataxia 5 s OR Spinocerebellar Ataxia-5 OR Spinocerebellar Ataxia Type 6 OR Type 6 Spinocerebellar Ataxia OR Spinocerebellar Ataxia 6 OR Spinocerebellar Ataxia 6 s OR Spinocerebellar Ataxia-6 OR Spinocerebellar Ataxia Type 1 OR Type 1 Spinocerebellar Ataxia OR Spinocerebellar Ataxia 1 OR Spinocerebellar Ataxia 1 s OR Spinocerebellar Atrophy I OR Spinocerebellar Atrophy Is OR SCA1 OR SCA1s OR Cerebelloparenchymal Disorder I OR Cerebelloparenchymal Disorder Is OR Menzel Type OPCA OR Schut-Haymaker Type OPCA OR Schut Haymaker Type OPCA OR Olivopontocerebellar Atrophy IV OR Olivopontocerebellar Atrophy IVs OR Spinocerebellar Ataxia-1 OR Olivopontocerebellar Atrophy I OR Olivopontocerebellar Atrophy Is OR Spinocerebellar Ataxia Type 4 OR Spinocerebellar Ataxia 4 OR Spinocerebellar Ataxia 4 s OR Spinocerebellar Ataxia-4 OR Type 4 Spinocerebellar Ataxia*
(I) Intervention	*Stem Cell OR Progenitor Cells OR Progenitor Cell OR Mother Cells OR Mother Cell OR Colony-Forming Unit OR Colony Forming Unit OR Colony-Forming Units OR Colony Forming Units OR Mesenchymal Stem Cell OR Bone Marrow Mesenchymal Stem Cells OR Bone Marrow Mesenchymal Stem Cell OR Bone Marrow Stromal Cells OR Bone Marrow Stromal Cell OR Multipotent Bone Marrow Stromal Cell OR Multipotent Bone Marrow Stromal Cells OR Adipose-Derived Mesenchymal Stem Cells OR Adipose Derived Mesenchymal Stem Cells OR Adipose Tissue-Derived Mesenchymal Stem Cell OR Adipose Tissue Derived Mesenchymal Stem Cell OR Adipose Tissue-Derived Mesenchymal Stem Cells OR Adipose Tissue Derived Mesenchymal Stem Cells OR Adipose Tissue-Derived Mesenchymal Stromal Cells OR Adipose Tissue Derived Mesenchymal Stromal Cells OR Adipose-Derived Mesenchymal Stromal Cells OR Adipose Derived Mesenchymal Stromal Cells OR Adipose-Derived Mesenchymal Stem Cell OR Adipose Derived Mesenchymal Stem Cell OR Mesenchymal Stromal Cells OR Mesenchymal Stromal Cell OR Multipotent Mesenchymal Stromal Cells OR Multipotent Mesenchymal Stromal Cell OR Mesenchymal Progenitor Cell OR Mesenchymal Progenitor Cells OR Wharton Jelly Cells OR Wharton’s Jelly Cells OR Wharton’s Jelly Cell OR Whartons Jelly Cells OR Bone Marrow Stromal Stem Cells*
(C) Control	*Any comparison*
(O) Outcome	*Function Recoveries OR Function Recovery*

Medline example: ((Cerebellar degeneration OR Spinocerebellar Ataxia OR Dominantly-Inherited Spinocerebellar Ataxias OR Dominantly Inherited Spinocerebellar Ataxias OR Dominantly-Inherited Spinocerebellar Ataxia OR Spinocerebellar Atrophies OR Spinocerebellar Atrophy OR Spinocerebellar Ataxia Type 2 OR Type 2 OR Spinocerebellar Ataxia OR Spinocerebellar Ataxia 2 OR Spinocerebellar Ataxia 2 s OR Wadia-Swami Syndrome OR Cerebellar Degeneration with Slow Eye Movements OR Olivopontocerebellar Atrophy II OR Olivopontocerebellar Atrophy IIs OR Spinocerebellar Atrophy II OR Spinocerebellar Atrophy IIs OR Olivopontocerebellar Atrophy 2 OR Olivopontocerebellar Atrophy 2 s OR Spinocerebellar Ataxia with Slow Eye Movements OR Spinocerebellar Atrophy 2 OR Spinocerebellar Atrophy 2 s OR Spinocerebellar Degeneration with Slow Eye Movements OR Wadia Swami Syndrome OR Spinocerebellar Ataxia-2 OR Spinocerebellar Ataxia Type 7 OR Type 7 Spinocerebellar Ataxia OR OPCA with Retinal Degeneration OR Olivopontocerebellar Atrophy III OR Olivopontocerebellar Atrophy IIIs OR Autosomal Dominant Cerebellar Ataxia, Type II OR OPCA with Macular Degeneration and External Ophthalmoplegia OR Spinocerebellar Ataxia-7 OR Spinocerebellar Ataxia 7 OR Spinocerebellar Ataxia 7 s OR Spinocerebellar Ataxia Type 5 OR Type 5 Spinocerebellar Ataxia OR Spinocerebellar Ataxia 5 OR Spinocerebellar Ataxia 5 s OR Spinocerebellar Ataxia-5 OR Spinocerebellar Ataxia Type 6 OR Type 6 Spinocerebellar Ataxia OR Spinocerebellar Ataxia 6 OR Spinocerebellar Ataxia 6 s OR Spinocerebellar Ataxia-6 OR Spinocerebellar Ataxia Type 1 OR Type 1 Spinocerebellar Ataxia OR Spinocerebellar Ataxia 1 OR Spinocerebellar Ataxia 1 s OR Spinocerebellar Atrophy I OR Spinocerebellar Atrophy Is OR SCA1 OR SCA1s OR Cerebelloparenchymal Disorder I OR Cerebelloparenchymal Disorder Is OR Menzel Type OPCA OR Schut-Haymaker Type OPCA OR Schut Haymaker Type OPCA OR Olivopontocerebellar Atrophy IV OR Olivopontocerebellar Atrophy IVs OR Spinocerebellar Ataxia-1 OR Olivopontocerebellar Atrophy I OR Olivopontocerebellar Atrophy Is OR Spinocerebellar Ataxia Type 4 OR Spinocerebellar Ataxia 4 OR Spinocerebellar Ataxia 4 s OR Spinocerebellar Ataxia-4 OR Type 4 Spinocerebellar Ataxia) AND (Stem Cell OR Progenitor Cells OR Progenitor Cell OR Mother Cells OR Mother Cell OR Colony-Forming Unit OR Colony Forming Unit OR Colony-Forming Units OR Colony Forming Units OR Mesenchymal Stem Cell OR Bone Marrow Mesenchymal Stem Cells OR Bone Marrow Mesenchymal Stem Cell OR Bone Marrow Stromal Cells OR Bone Marrow Stromal Cell OR Multipotent Bone Marrow Stromal Cell OR Multipotent Bone Marrow Stromal Cells OR Adipose-Derived Mesenchymal Stem Cells OR Adipose Derived Mesenchymal Stem Cells OR Adipose Tissue-Derived Mesenchymal Stem Cell OR Adipose Tissue Derived Mesenchymal Stem Cell OR Adipose Tissue-Derived Mesenchymal Stem Cells OR Adipose Tissue Derived Mesenchymal Stem Cells OR Adipose Tissue-Derived Mesenchymal Stromal Cells OR Adipose Tissue Derived Mesenchymal Stromal Cells OR Adipose-Derived Mesenchymal Stromal Cells OR Adipose Derived Mesenchymal Stromal Cells OR Adipose-Derived Mesenchymal Stem Cell OR Adipose Derived Mesenchymal Stem Cell OR Mesenchymal Stromal Cells OR Mesenchymal Stromal Cell OR Multipotent Mesenchymal Stromal Cells OR Multipotent Mesenchymal Stromal Cell OR Mesenchymal Progenitor Cell OR Mesenchymal Progenitor Cells OR Wharton Jelly Cells OR Wharton’s Jelly Cells OR Wharton’s Jelly Cell OR Whartons Jelly Cells OR Bone Marrow Stromal Stem Cells)) AND (Function Recoveries OR Function Recovery)

### Other research sources

In an effort to identify additional published, unpublished and ongoing trials, we performed the following steps:

- screened the reference lists of the identified studies;
- contacted the study authors and experts; and.
- used the Science Citation Index Cited Reference Search to track important articles.

### Studies selection

Studies with individuals diagnosed with spinocerebellar ataxia undergoing treatment with stem cells were included, with outcome endpoints such as motor function, language disorders, ocular motility disorders, quality of life, static and gait balance and treatment safety. The following were excluded: duplicate articles; systematic reviews; unavailable in full articles, chapters or abstracts; animal or cell-based models; case studies or series case; case-control; cross-sectional studies; cohort studies and off topics. Two pairs of reviewers independently screened all titles and abstracts identified in the literature search, obtained full-text articles of all the potentially eligible studies, and evaluated them for eligibility. The reviewers resolved disagreements by discussion or, if necessary, with third party adjudication. We also considered studies reported only as conference abstracts.

### Data extraction

The reviewers underwent calibration exercises and worked in pairs to independently extract data from the included studies according to the recommendations of the Cochrane Handbook for Systematic Reviews of Interventions [17]. They resolved disagreements by discussion or, if necessary, with third party adjudication. They abstracted the following data using a pretested data extraction form: study design, participants, interventions, outcomes assessed, follow-up and relevant statistical data.

### Bias risk assessment

Two authors of this review independently assessed the risk of bias for each study using the criteria outlined in the Cochrane Handbook for Systematic Reviews of Interventions. We resolved disagreements by discussion or by consultation with another review author. We assessed the risk of bias according to the following domains.

- Random sequence generation.
- Allocation concealment.
- Blinding of the participants and personnel.
- Blinding of the outcome assessment.
- Incomplete outcome data.
- Selective outcome reporting.
- Other bias.

We graded the risk of bias for each domain as high, low, or unclear and provided information from the study report, together with justification for our judgment, in the “Risk of bias” table. For incomplete outcome data in individual studies, we stipulated a low risk of bias for a loss to follow-up of less than 10% and a difference of less than 5% in missing data between the intervention/exposure and control groups.

### Evidence recommendation

We summarized the evidence and assessed its certainty separately for bodies of evidence from Randomized Controlled Trials (RCT) and non-RCT studies. We used the Grading of Recommendations Assessment, Development and Evaluation (GRADE) methodology to rate the certainty of the evidence for each outcome as high, moderate, low, or very low. In the GRADE approach, RCTs begin with high certainty, and non-RCT studies begin with moderate certainty. Detailed GRADE guidelines were used to assess the overall risk of bias, imprecision, inconsistency, indirectness, and publication bias and to summarize the results in an evidence profile (Table 3) [22].

We planned to assess publication bias through the visual inspection of funnel plots for each outcome for which we identified 10 or more eligible studies; however, we were not able to do so because there were an insufficient number of studies to conduct this assessment.

### Data synthesis and statistical analysis

All outcomes as continuous variables were analyzed. The results were presented as mean of differences (MD) along with 95% confidence intervals, using fixed-effects models. The unit of analysis was each participant recruited for review. The variability in results across studies was checked by using the I^2^statistic and the *p*-value for the chi square test of heterogeneity provided by Review Manager. In addition, Review Manager (RevMan) (version 5.3; Nordic Cochrane Centre, Cochrane) was used for all analyses. Due to the small number of studies that were identified, sensitivity tests (e.g., low versus high risk of bias) were not performed and subgroups were not applied.

## Results

**Fig. 1:**
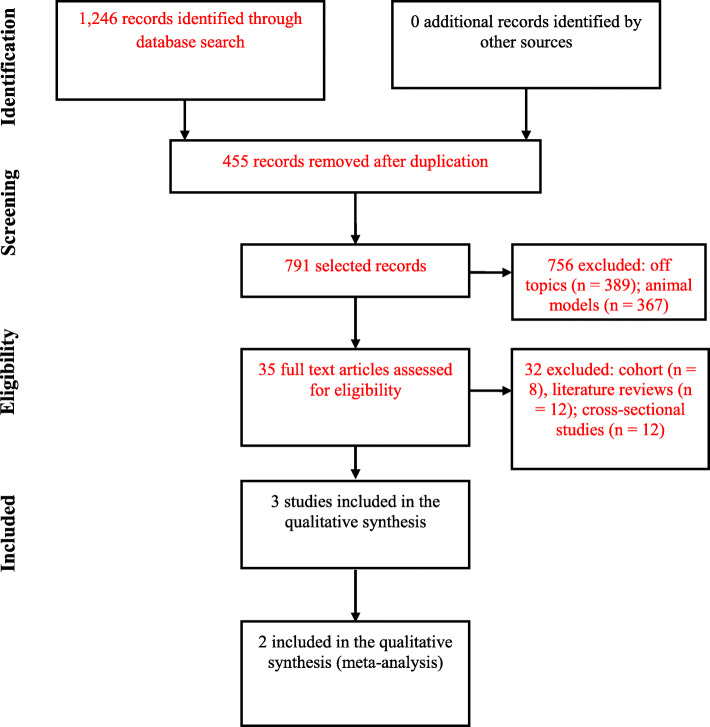
Flowchart for article selection

We identified a total of 143 studies (401 of which were from the PUBMED database, 62 from Bireme, 779 from Science Direct, 1 from Cochrane and 3 from Clinical Trials). After screening the titles and then the abstracts, we obtained full-text articles for the 27 studies that were potentially eligible for inclusion in the review. We excluded 24 studies because they were considered observational studies. This left 3 clinical trials [19–21] for analysis and 2 [19, 20] for meta-analysis based on homogeneous outcomes. The others were excluded because they were prospective studies, literature reviews and cross-sectional studies (Fig. 1).

The main characteristics of the three selected studies are showed in the Table 2. In all included studies did not have adverse effects.

**Table 2:** Characteristics of the included studies in the review

Authors (year)	Participants	Study Design	Country	Inclusion Criteria	Exclusion Criteria	Outcome assessed	Follow-up	PEDro Score
**Dongmei et al. (2011)** **[**19**]**	*n* = 24 (14: SCA and 10: MSA-C) Age: 46 years old	Non-randomized clinical trial	China	SCA	Cardiopulmonary, renal and hepatic dysfunction, or systemic infection.	ICARS and ADL	6 and 15 months	5 Points
**Jin et al. (2013)** **[**20**]**	*n* = 16 Age: 39.9 ± 10.2 years old	Open label uncontrolled clinic trial	China	SCA1, SCA2 or SCA3, age 16–65 years old.	Cardiopulmonary, renal and hepatic dysfunction, or systemic infection; tumor markers; blood pressure > 180/110 mmHg; pregnancy or breastfeeding.	BBS and ICARS	3, 6 and 12 months	6 Points
**Tsai et al. (2017)** **[**21**]**	*n* = 7 Age: 48.85 ± 14.75 years old	Pilot open label Phase I / IIa clinical trial	Taiwan	SCA 3 or MSA-C varying 10–20 on SARA	Another trial with cell therapy less than 30 days, positive pregnancy test.	SARA, SOT, MRI and F-FDG PET	12 months	5 Points

Legends: *SCA* Spinocerebellar Ataxia, *MSA-C* Multiple System Atrophy-Cerebelar Type, *SARA* Scale for the Assessment and Rating of Ataxia, *ICARS* International Cooperative Ataxia Rating Scale, *ADL* Activity of Daily Living Scale, *BBS* Berg Balance Scale, *SOT* Posturography, *MRI* Magnetic Resonance Imaging, *F-FDG PET* Fluoro-D-glucose integrated with computed tomography. The diagnosis of spinocerebellar ataxia in all studies followed Harding’s diagnostic pattern with molecular confirmation

The interventions, results and GRADE for each selected study are showed in the Table 3. All studies displayed low certainty in estimates or quality of evidence.

**Table 3:** Interventions, results and GRADE of studies included in the review

Author/year	Interventions	Relevant statistical data	Grade
Dongmei et al., 2011 [19]	UCMSCs (1 × 10^6^ /kg; 10 mL) with 5 mg dexamethasone was injected intrathecally weekly for 4 weeks by lumbar punctures, resulting in 4 injections for 4 weeks.	ICARS = ↑ posture, gait disorders, coordination, speech disorders, ocular motility. ADL = ↑ self-care ability.	⊕ ⊕ ϴϴ Low
Jin et al., 2013 [20]	UCMSCs (4 × 10^7^) in 30 ml saline solution IV in 30 min and 3 treatments, 2 × 107 in 30 ml IV and 2 × 107 in 1 ml IT simultaneously. 4 applications a week apart.	After the first application of UCMSCs, 38% (6/16) ↑ BBS. From 3 to 6 months 63% (10/16) ↑ BBS and there were statistically significant improvements over the baseline. After 1-year 7/16 (44%) ↑ BBS over the baseline, and only 5/16 (31%) of the patients suffered from the disease. ↑ ICARS in the 3rd and 6th month.	⊕ ⊕ ϴϴ Low
Tsai et al., 2017 [21]	100 g of adipose tissue was collected by liposuction of the abdominal region of two healthy donors. The AD-MSC were frozen at a concentration of 7 × 107 viable cells in 20 ml of cryopreservation solution. On the day of infusion, thawed AD-MSCs were mixed with 100 ml with normal saline and administered IV in 40 min.	SARA score evolution: 13.25 at the baseline, 13.0 in the first 15 days, 12.75 at 6 months, and 13.5 at 12 months.	⊕ ⊕ ϴϴ Low

*UCMSC* Umbilical cord mesenchimal stem cells, *ICARS* Intarnational Cooperative Ataxia Rading Scale, *ADL* Activity of Daily Living Scale, *IT* Intrathecal, *BBS* Berg Balance Scale, *AD-MSCs* Adipose tissue mesenchimal stem cells, *IV* Intravenous, *SARA* Scale for the Assessment and Randing of Ataxia

For the random sequence generation, all studies demonstrated high risk of bias: Dongmei et al. (2011) [19] is a non-randomized clinical trial; Jin et al. (2013) [20] is an open label uncontrolled clinical trial and Tsai et al. (2017) [21] is a pilot open label phase I/IIa clinical trial. The allocation concealment and blinding of the participants and personnel also presented a high risk of bias for all studies. Regarding the blinding of outcome assessment, Dongmei et al. (2011) [19] and Jin et al. (2013) [20], presented low risk of bias, because they used outcome evaluators during follow-up, and Tsai, et al. (2017) [21] does not report this information. All studies presented low risk of bias in the incomplete outcome data and selective outcome reporting.

The risk of bias of the included studies is shown in Table 4.

**Table 4:** Risk of Bias Classification

Risk of Bias	High Risk	Low Risk	Uncertain Risk
Random sequence generation	Dongmei et al., 2011 [19] Jin et al., 2013 [20] Tsai et el., 2017 [21]	None	None
Allocation concealment	Dongmei et al., 2011 [19] Jin et al., 2013 [20] Tsai et el., 2017 [21]	None	None
Blinding of participants and professionals	Dongmei et al., 2011 [19] Jin et al., 2013 [20] Tsai et el., 2017 [21]	None	None
Blinding of outcome evaluators	None	Dongmei et al., 2011 [19] Jin et al., 2013 [20]	Tsai et el., 2017 [21]
Incomplete outcomes data	None	Dongmei et al., 2011 [19] Jin et al., 2013 [20] Tsai et el., 2017 [21]	None
Selective outcome reporting	None	Dongmei et al., 2011 [19] Jin et al., 2013 [20] Tsai et el., 2017 [21]	None

The risk of bias was graded for each domain as high, low, or unclear. For incomplete outcome data in individual studies, we stipulated a low risk of bias for a loss to follow-up of less than 10% and a difference of less than 5% in missing data between the intervention/exposure and control groups

**Fig. 2:**
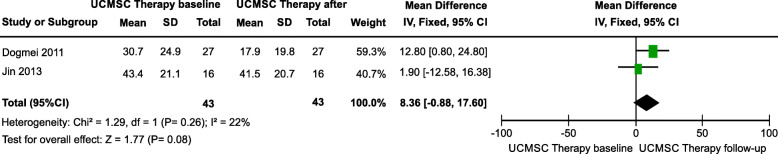
Meta-analysis of included studies (outcome: ICARS)

The meta-analysis was performed with two studies due to the variability of the outcomes. The included studies in the meta-analysis were: Dongmei et al. (2011) [19], who injected intrathecally UCMSCs and Jin et al. (2013) [20], who performed IV injections of Umbilical Cord Mesenchymal Stem Cells (UCMSCs). For these studies, the outcome included in the meta-analysis was the ICARS scale score. Figure 2 shows the meta-analysis of Dongmei, 2011 [19] and Jin, 2013 [20] studies. We observed that there is no statistically significant difference (MD = 8.36, 95% CI, 0.88, 17.60; *p* = 0.08) between the stem cell groups in the baseline and follow-up in both studies.

## Discussion

Based on the results of the meta-analysis we observed that there are no statistically significant differences in the ICARS scale score before and after the application of stem cells in SCA considering the two included studies. The ICARS was developed by Trouillas et al. (2011) [23] and comprises 19 items, divided in four subscales: 1) posture and gait disturbances (items 1–7, score 0–34); 2) kinetic functions (items 8–14, score 0–52); 3) speech disorders (items 15–16, score 0–8); and 4) oculomotor disorders (items 17–19, score 0–6), along with a functional test (Archimedes spiral). The maximum possible score is 100. The Minimal Clinical Importance Difference (MICD) of ICARS above 2 shows clinical and functional improvement [24], and in our study the improvement in score was 8.36, and we can infer that individuals with SCA submitted to stem cell treatment, even without significant results in the meta-analysis, showed significant clinical improvement in the functional recovery.

Because they are not randomized clinical trials, there is no concealment of allocation or blinding of participants or evaluators and even if there is a report on outcome evaluators, justification of loss and exclusion of participants and availability of protocols, following the criteria of the GRADE system, there is a low evidence recommendation for the use of stem cell protocol in spinocerebellar ataxia. In the studies included in this review, there were heterogeneous study designs and small sample size, which can be explained by the fact that it is a rare disease, leading to a low number of participants, thus influencing the results of the meta-analysis. If there are studies with different designs than the studies included in this review, such as randomization, blinding, among others; the recommendation for evidence will be higher.

Through this systematic review, we can observe the clinical efficacy and safety of treatments involving individuals with spinocerebellar ataxia who have undergone stem cell treatments, as well as other types of ataxia, such as multiple systems atrophy-cerebellar type. In all studies, research participants had no major side effects.

Among the various sources for stem cell extraction, two studies used cells from umbilical cord [19, 20] and one study used cells from adipose tissue [21]. In the study by Dongmei et al. (2011) [19], there was a significant improvement in the ICARS and ADL (Activity of Daily Living Scale) scales, in addition to no adverse effects. Even though in some cases, the progression of the disease has not been prevented, there was an observed delay in the degenerative process, in addition to an increase in the time of stabilization of the disease. Jin et al. (2013) [20], also showed improvement in the ICARS and Berg Balance Scale, mainly from 3 to 6 months after application of stem cells from the umbilical cord. Tsai et al. (2017) [21] showed that applications with cells from adipose tissue, show little significant results as to their effectiveness, but they were shown to be safe, evaluating individuals with the SARA scale, posturography and magnetic resonance imaging.

Interestingly, when we evaluated the studies separately, we can see that there were improvements in the motor parameters of individuals with spinocerebellar ataxia undergoing stem cell therapy, but when evaluated in the meta-analysis, the result was influenced by the small sample size and the high variability of the outcomes. Furthermore, knowing the age variability presented in the included studies and the difference in the cell sources used for each procedure, some questions remain unanswered for future work. What is the difference in the effectiveness of stem cell treatment in individuals with spinocerebellar ataxia at different ages? Are the results similar in younger and older people? Stem cells extracted from which source have the best effects? Does variability in isolation of cells affect outcomes? Which cells are more efficient, autologous or allogeneic?

### Strengths and limitation

Strengths of our review include a comprehensive search; assessment of eligibility, risk of bias, and data abstraction independently and in duplicate; assessment of risk of bias that included a sensitivity analysis addressing loss to follow-up; and use of the GRADE approach for rating the certainty of evidence for each outcome. Furthermore, there were no language restrictions, and translations of non-English trials were obtained whenever possible. The primary limitation of our review is the low certainty consequent to study limitations. We identified a small number of RCTs with a modest number of participants resulting in wide confidence intervals. The total number of participants was relatively very low due to the small sample sizes of individual trials, which led to downgrading the quality of evidence in some instances because underpowered trials are likely to have a greater degree of imprecision. Moreover, selection bias and unblinding were substantial. Another limitation of this review was having an insufficient number of included studies to allow for the complete statistical analysis that we had planned. We were not able to assess publication bias because there were fewer than 10 eligible studies addressing the same outcome in a meta-analysis.

### Implications

Low-quality evidence shows that steam cell therapy is more efficacious for functional recovery after SCA measured by ICARS. Future trials should adhere to CONSORT guidelines to ensure clarity and reproducibility in the reporting of methods. There are no specific systematic reviews on stem cell treatments for patients with spinocerebellar ataxia, demonstrating the importance of this review, in order to achieve a greater understanding by researchers, health professionals and patients on the subject. This review can assist future research, as it brings together important data regarding the target audience, type of cells used, form of application, evaluation criteria, among others. There is a need for more research related to this therapy with careful designs, such as randomized, blind or double-blind clinical trials with a larger sample size and less risk of bias.

## Conclusion

There was low evidence for recommending stem cell therapy in individuals with spinocerebellar ataxia, and no statistical difference was observed for improving functional recovery of patients. In addition, it should be taken into account that the studies included in this review present risks of bias and methodological flaws, and therefore, it is recommended to develop clinical trials of larger sample size and lower risk of bias so that future conclusions can be based on more robust searches.

## Data Availability

Not applicable.
